# Publisher Correction: Clonal replacement and heterogeneity in breast tumors treated with neoadjuvant HER2-targeted therapy

**DOI:** 10.1038/s41467-019-10456-x

**Published:** 2019-05-30

**Authors:** Jennifer L. Caswell-Jin, Katherine McNamara, Johannes G. Reiter, Ruping Sun, Zheng Hu, Zhicheng Ma, Jie Ding, Carlos J. Suarez, Susanne Tilk, Akshara Raghavendra, Victoria Forte, Suet-Feung Chin, Helen Bardwell, Elena Provenzano, Carlos Caldas, Julie Lang, Robert West, Debu Tripathy, Michael F. Press, Christina Curtis

**Affiliations:** 1 Department of Medicine, Division of Oncology, Stanford University School of Medicine, Stanford, 94305 California United States; 2 Department of Genetics, Stanford University School of Medicine, Stanford, 94305 CA USA; 3 Stanford Cancer Institute, Stanford University School of Medicine, Stanford, 94305 CA USA; 4 Canary Center for Cancer Early Detection, Department of Radiology, Stanford University School of Medicine, Palo Alto, 94305 CA USA; 5 Department of Pathology, Stanford University School of Medicine, Stanford, 94305 CA USA; 6 Department of Biology, Stanford University, Stanford, 94305 CA USA; 7 Department of Breast Medical Oncology, The University of Texas MD Anderson Cancer Center, Houston, 77030 TX USA; 8 Maimonides Medical Center, Brooklyn, 11219 NY USA; 9 Norris Comprehensive Cancer Center, Los Angeles, 90033 CA USA; 10 Cancer Research UK Cambridge Institute, Department of Oncology, University of Cambridge, Cambridge, CB2 0RE UK; 11 Cambridge Experimental Cancer Medicine Centre and NIHR Cambridge Biomedical Research Centre, Cambridge University Hospitals NHS Foundation Trust, Cambridge, CB2 0QQ UK; 12 Department of Surgery, Keck School of Medicine, University of Southern California, Los Angeles, 90333 CA USA; 13 Department of Pathology, Keck School of Medicine, University of Southern California, Los Angeles, 90033 CA USA

**Keywords:** Breast cancer, Cancer genomics

Correction to: *Nature Communications* 10.1038/s41467-019-08593-4, published online 08 February 2019.

The original version of this Article omitted from the Author Contributions statement that ‘R.S. and J.G.R. contributed equally to this work.’ This has been corrected in both the PDF and HTML versions of the Article.

