# Supplementary material for: Clonal replacement and heterogeneity in breast tumors treated with neoadjuvant HER2-targeted therapy
Source: Nat Commun. 2019 Feb 8;10:657. doi: 10.1038/s41467-019-08593-4 (PMC6368565; doi:10.1038/s41467-019-08593-4)
Supplement: Supplementary file 1 — Supplementary Information [file 41467_2019_8593_MOESM1_ESM.pdf]

# Supplementary Information

# Clonal replacement and heterogeneity in breast tumors treated with neoadjuvant HER2-targeted therapy

Caswell-Jin et al.

## Supplementary Figures

Supplementary Figure 1. Measures of intra-tumor heterogeneity

Supplementary Figure 2. Inference of primary breast tumor growth parameters

Supplementary Figure 3. Intra-tumor heterogeneity across simulated deme sizes

Supplementary Figure 4. Value of additional samples in reclassifying clonal mutations as subclonal

Supplementary Figure 5. Flowchart describing the selection of high-quality tumors for analysis

Supplementary Figure 6. Intra-tumor heterogeneity in untreated vs treated primary breast tumors

Supplementary Figure 7. Inferred mutation clusters across diagnostic and surgical samples

Supplementary Figure 8. Sampling schema for the virtual tumors

Supplementary Figure 9. Information about precise sampling scheme can improve ability to assess for clonal evolution

Supplementary Figure 10. Increased post-treatment copy number similarity after clonal replacement

Supplementary Figure 11. Somatic mutational signatures across mutation groupings

## Supplementary Tables

Supplementary Table 1. Percentage of mutations identified as clonal in the first region that were later determined to be rare in additional tumor regions

Supplementary Table 2. Characteristics of analyzed and excluded treated HER2+ tumors

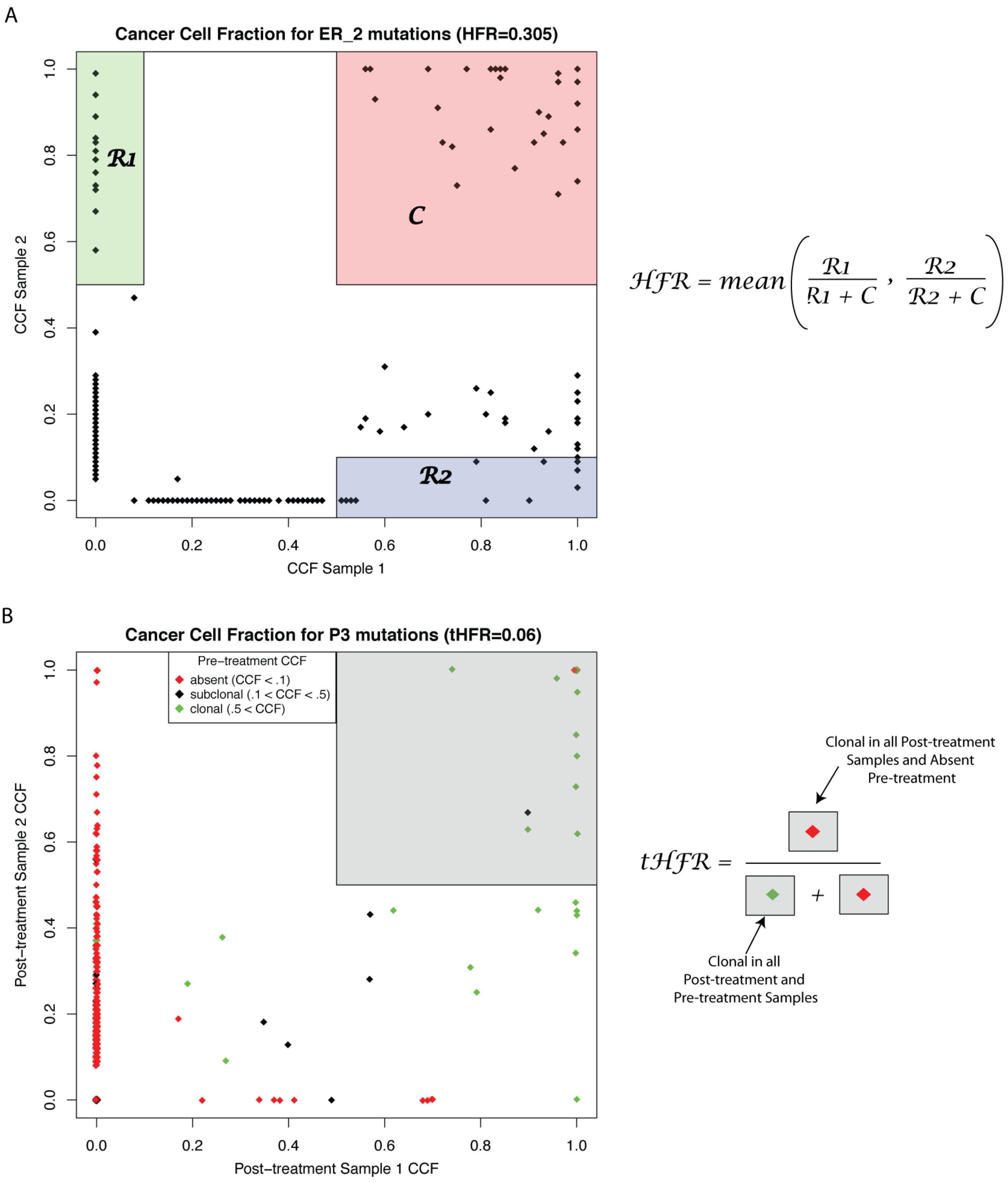


**Supplementary Figure 1. Measures of intra-tumor heterogeneity (a)** HFR (high-frequency regional). Clonal mutations *C* are defined as those with CCF > 0.5 in both samples; regional mutations *R1* and *R2* are those mutations present at CCF > 0.5 in one sample and CCF < 0.1 in the other sample. **(b**) tHFR (temporal high-frequency regional) is calculated across time, with one sample from one timepoint (e.g. pre-treatment) and one or more samples from another timepoint (e.g. post-treatment). Red diamonds represent mutations with CCF > 0.5 in all samples from the second timepoint and CCF < 0.1 in the sample from the first timepoint, while green diamonds represent mutations with CCF > 0.5 in all samples.

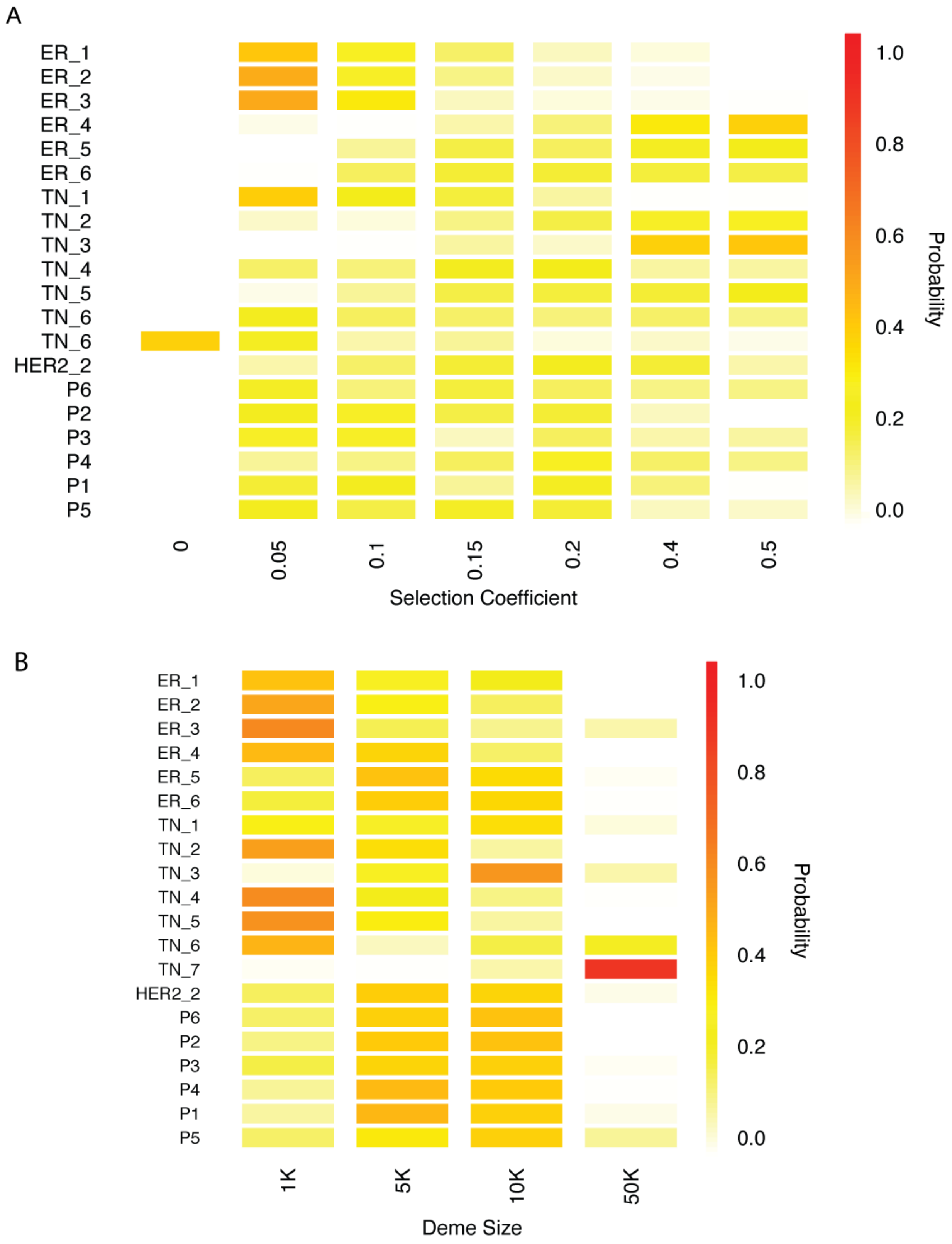


**Supplementary Figure 2. Inference of primary breast tumor growth parameters (a)** Heatmap showing, for each patient tumor, the posterior probability distribution of the selection coefficient inferred via Approximate Bayesian Computation. Nineteen of twenty tumors, across breast cancer subtypes and both treated and untreated, were inferred to have grown under strong selection ($s > 0.05$). **b**. Heatmap showing, for each patient tumor, the distribution of deme sizes inferred using the simulated tumors. A large deme size (50,000 cells) was rarely inferred, but deme sizes 1,000-10,000 were plausible. Source data are provided as a Source Data file.

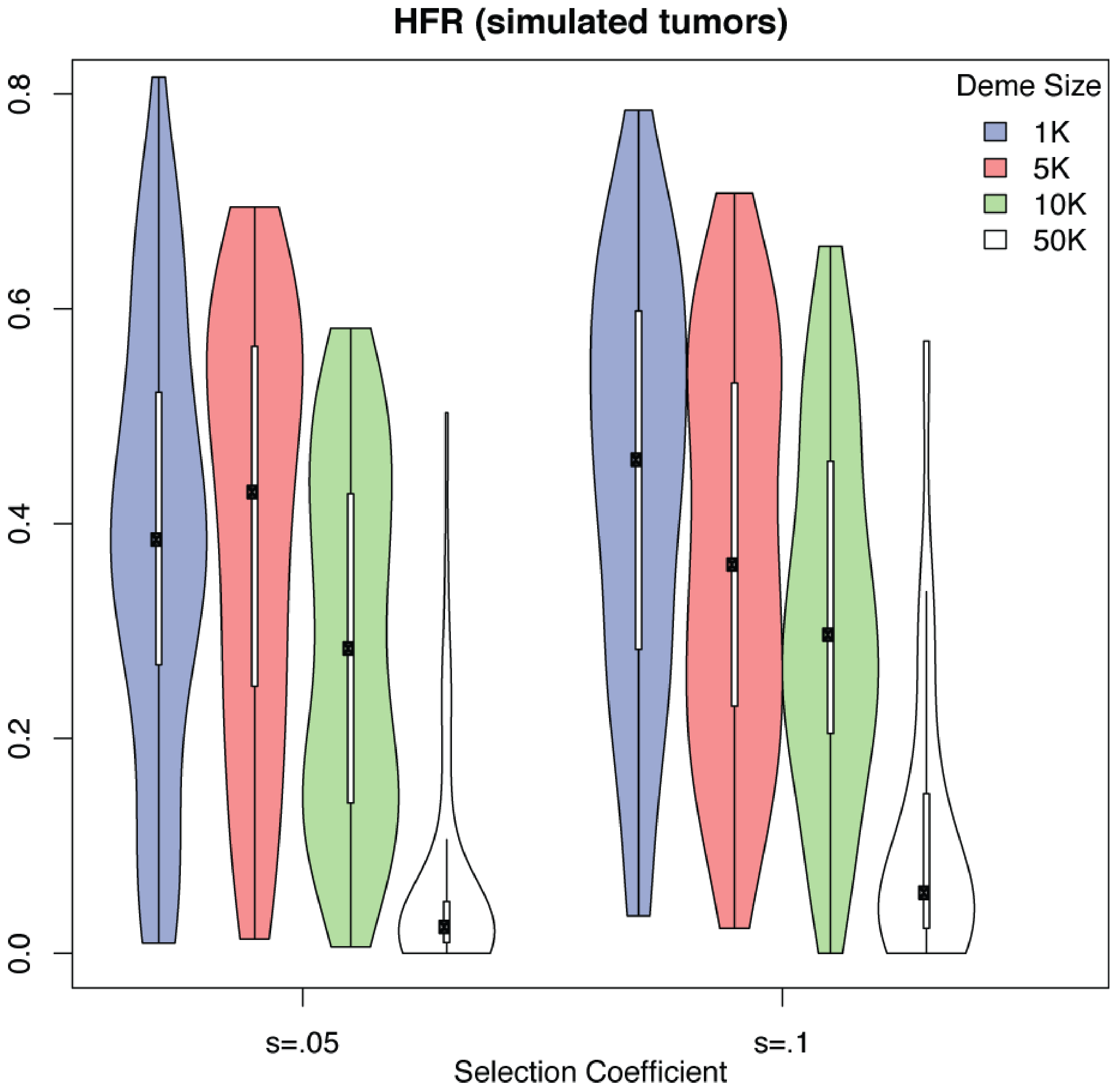


**Supplementary Figure 3. Intra-tumor heterogeneity across simulated deme sizes** Simulated tumors that grew with larger deme sizes tended to exhibit lower heterogeneity as measured with HFR (high-frequency regional) than those that grew with smaller deme sizes. This effect was greatest at a deme size of 50,000, which was statistically unlikely to be consistent with the primary breast tumors based on Approximate Bayesian Computation. Source data are provided as a Source Data file.

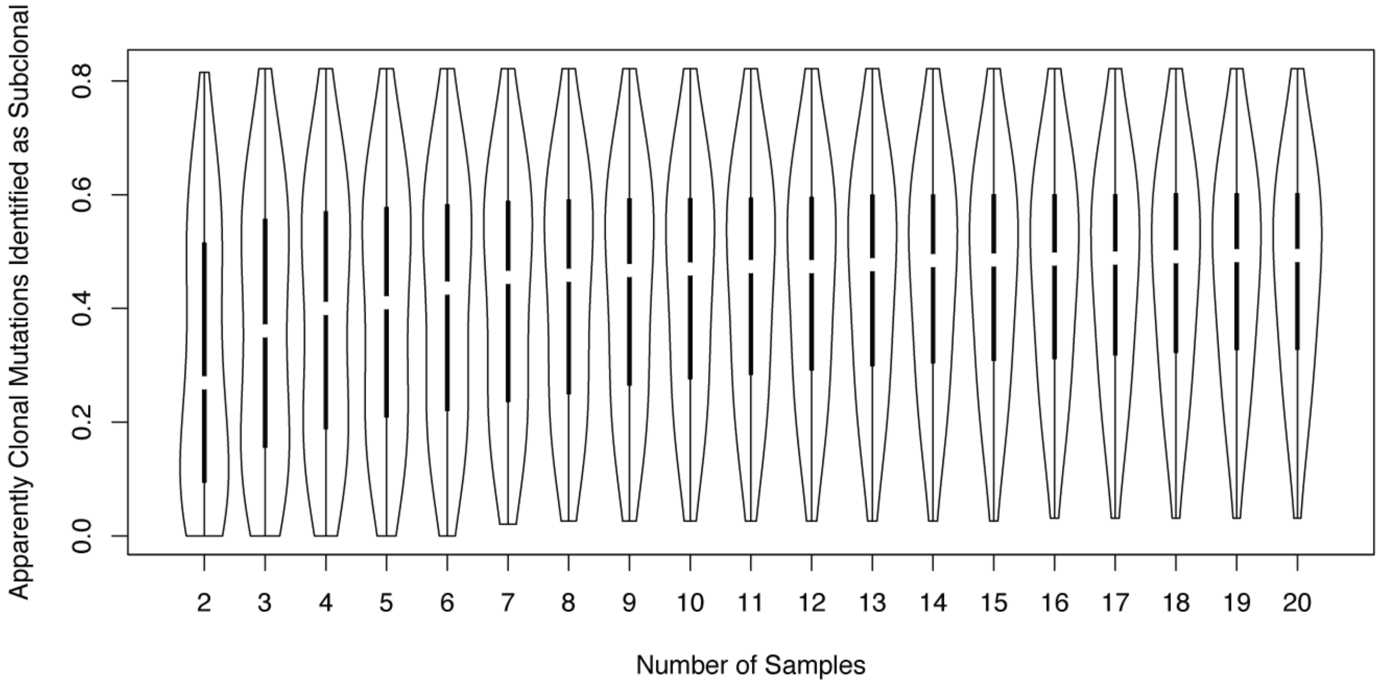


**Supplementary Figure 4. Value of additional samples in reclassifying clonal mutations as subclonal** Distribution of the proportion of apparently clonal mutations in one region reclassified as subclonal upon sampling 1-19 additional regions at random, for tumors that were simulated under varied selection coefficients (*s* = 0.05; 0.1; 0.2; 0.4; 0.5) consistent with primary breast tumor growth. Simulations under different non-neutral selection coefficients were combined given similarity of results. Great variability was seen between tumors. Source data are provided as a Source Data file.

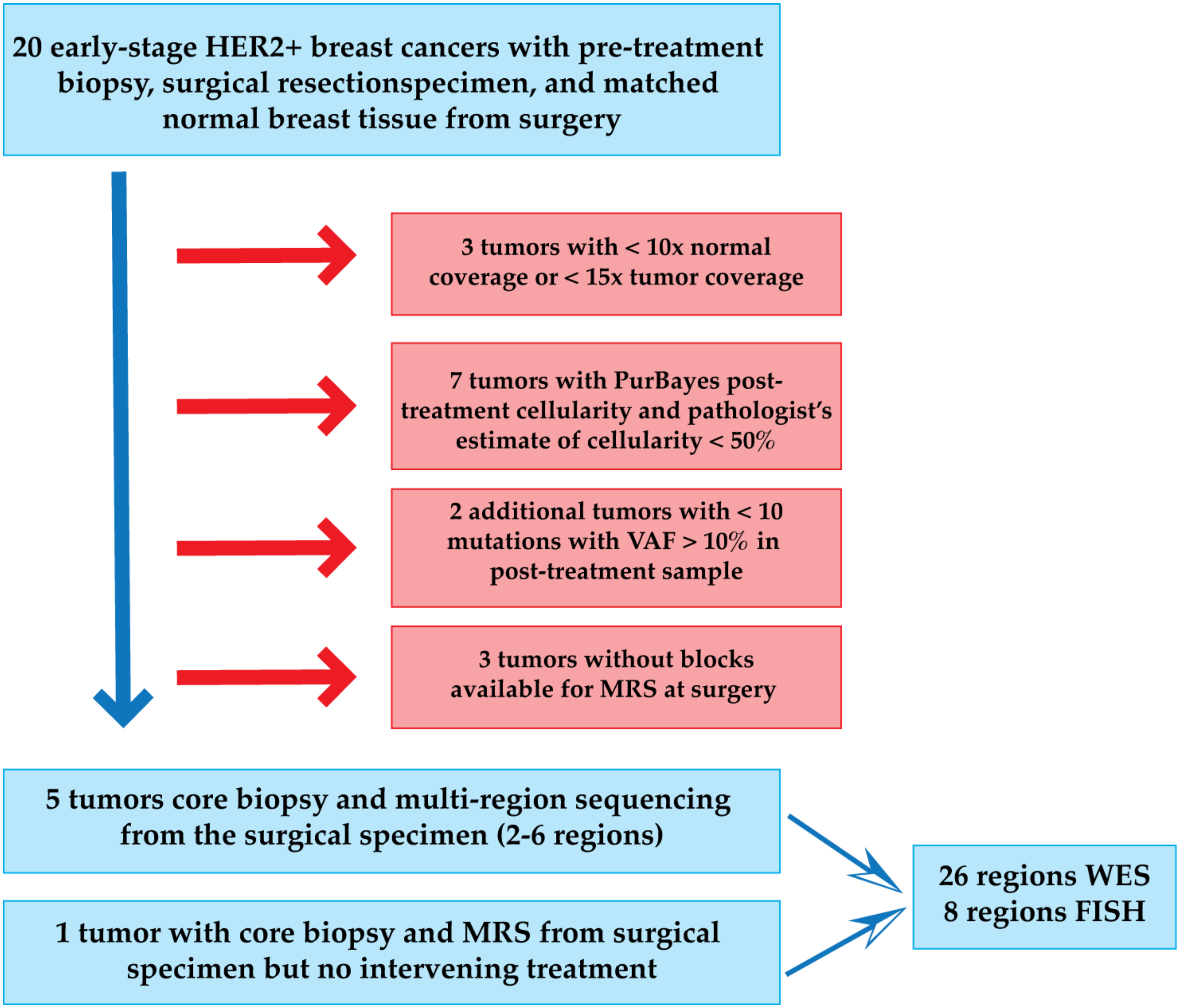


**Supplementary Figure 5. Flowchart describing the selection of high-quality tumors for analysis** Note that of the 9 tumors excluded based on poor post-treatment cellularity (steps 2 and 3 in the flowchart), 2 would also have been excluded based on poor pre-treatment cellularity.

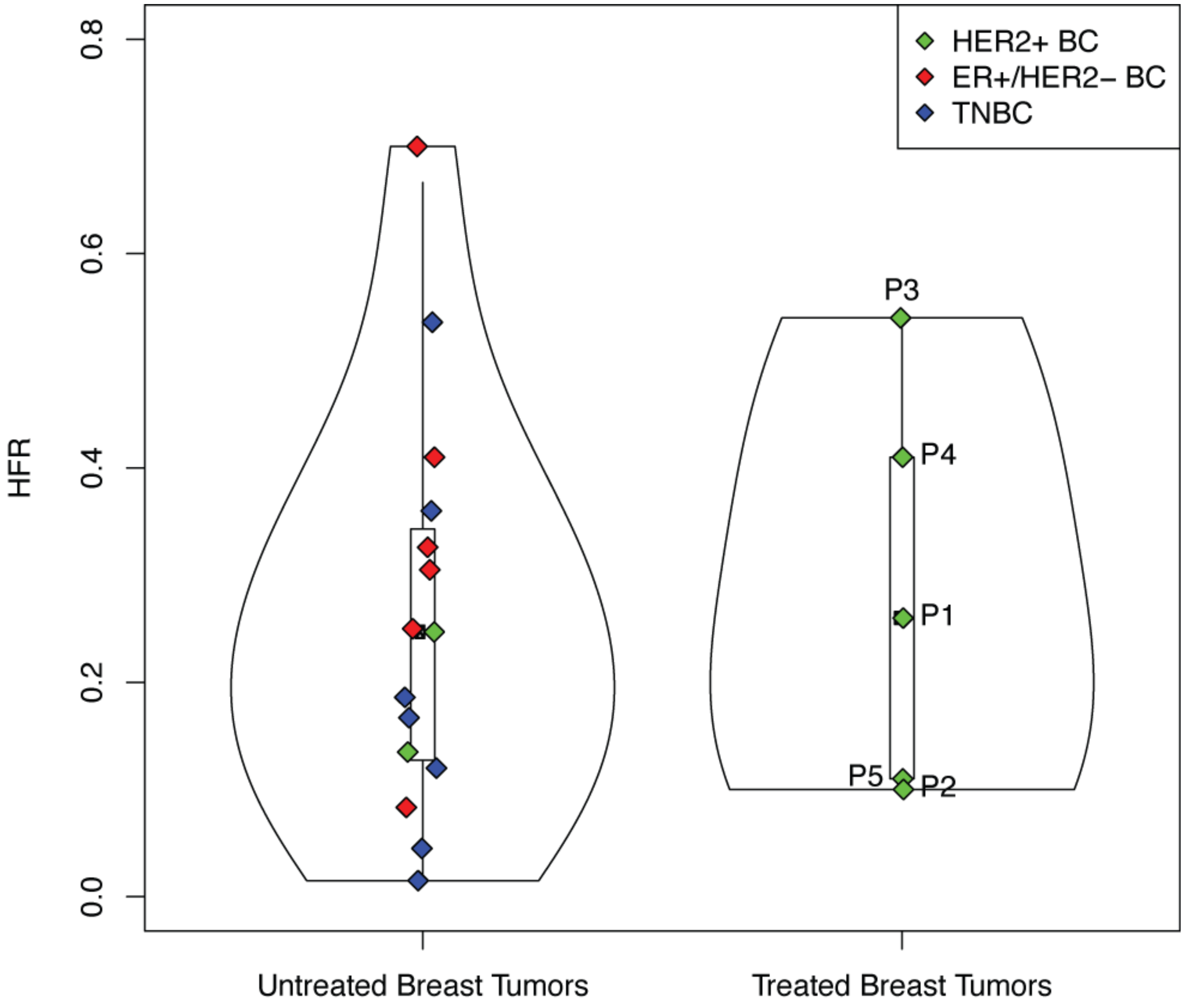


**Supplementary Figure 6. Intra-tumor heterogeneity in untreated vs treated primary breast tumors** HFR = high-frequency regional, BC = breast cancer, TN = triple-negative. Source data are provided as a Source Data file.

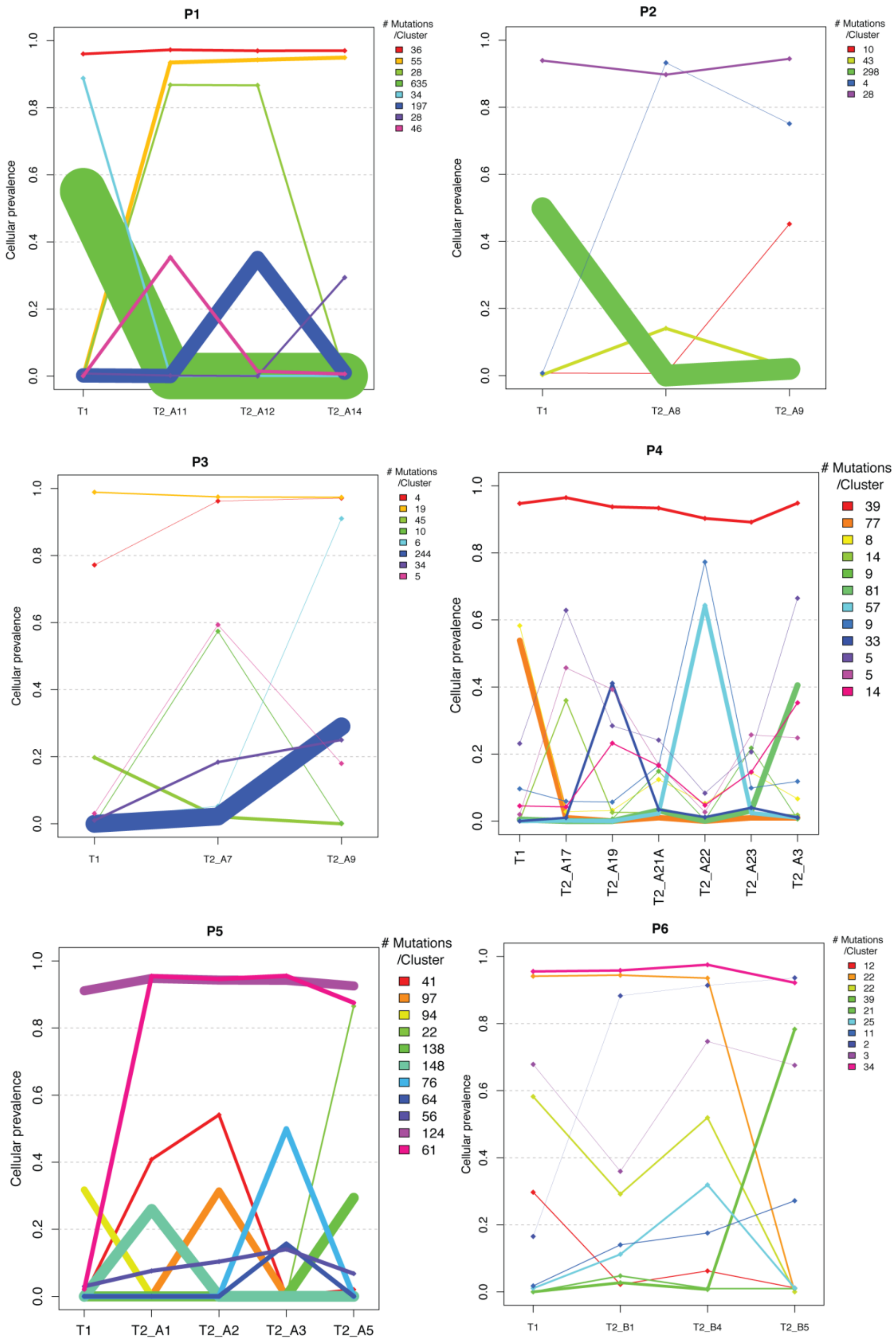


**Supplementary Figure 7. Inferred mutation clusters across diagnostic and surgical samples**
PyClone was used to define mutational clusters and assess changes in cluster frequencies across samples. P1-P5 were treated between the diagnostic timepoint (T1) and the surgical timepoint (T2), while P6 was not. In P1 and P5, a cluster of high frequency mutations was observed in all post-treatment regions that was absent or rare in the pre-treatment region. Multiple other mutation clusters were seen to be present in only one region at low or high frequency, or to be absent in only one region. This pattern of heterogeneity was seen in both the treated and untreated tumors. Source data are provided as a Source Data file.

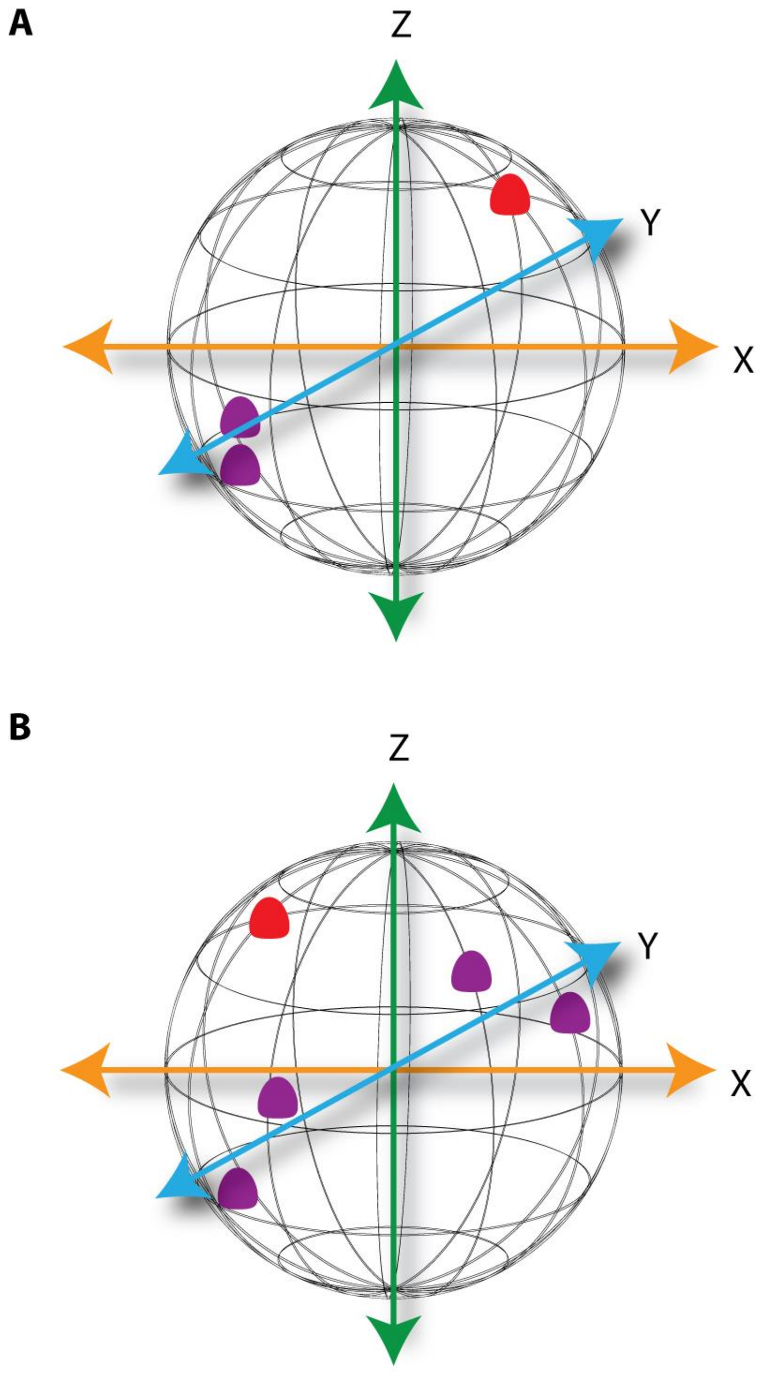


**Supplementary Figure 8. Sampling schema for the virtual tumors** The virtual tumors exist on a 3D lattice, with each subpopulation of cells (i.e. deme) having an explicit location relative to the *x*, *y*, and *z* axes. The eight octants are subspaces of the tumor defined by all possible combinations of positive and negative values for *x*, *y*, and *z* (e.g. octant 1 contains the tumor cells with $x>0$, $y>0$, and $z>0$ while the cells in octant 8 have $x<0$, $y<0$, $z<0$). The red dots indicate the pre-treatment sample, while the purple dots indicate the spatial distribution of post-treatment samples. **(a)** The general sampling scheme used to generate estimates of tHFR. The two post-treatment samples come from the same octant while the pre-treatment sample is from the most distant octant (with opposite signs for *x*, *y*, and *z*). **(b)** The virtual sampling scheme used to generate estimates of tHFR for P5 (where specific information about sampling scheme is known). In this case two post-treatment samples come from one quadrant and the other two post-treatment samples come from the opposite quadrant most distant from the first. The pre-treatment sample location is randomly chosen from all octants.

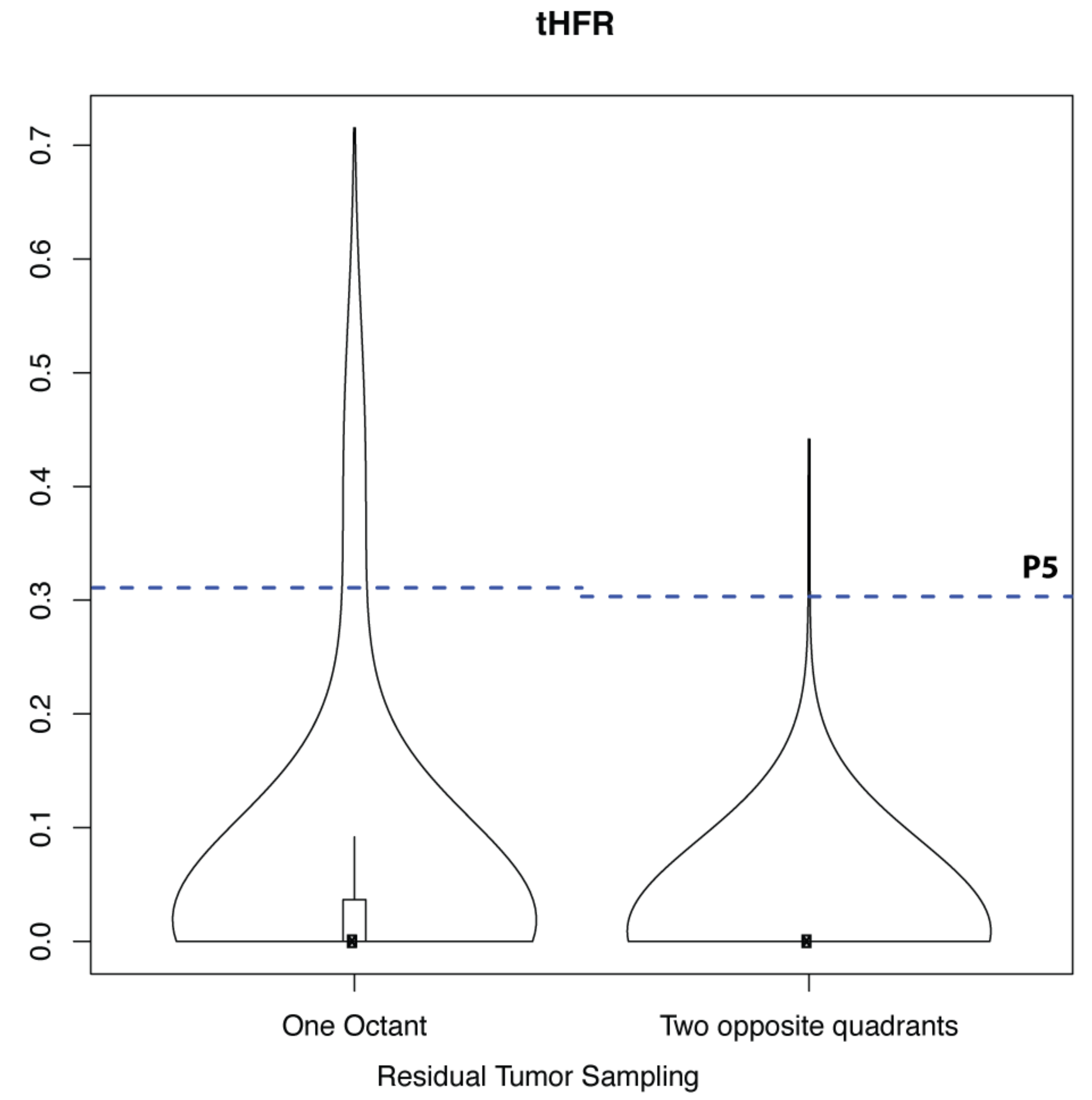


**Supplementary Figure 9. Information about precise sampling scheme can improve ability to assess for clonal evolution** In P5, the two pairs of post-treatment samples came from opposite quadrants of the tumor. We used this sampling scheme to sample from virtual (simulated) tumors in order to generate a more specific distribution of tHFR values for this case. Here we plot the tHFR distributions for the general sampling scheme with three samples from the same octant and for the more precise sampling scheme outlined in Supplementary Figure 8B. The blue dotted lines correspond to the tHFR values for P5 computed with three (all possible subsamples of three regions) and then four post-treatment regions. These findings demonstrate that the degree of clonal change seen in P5 was statistically unlikely to have resulted from pre-existing heterogeneity (tHFR > 99$^{th}$ percentile) under the four-sample precise sampling scheme. Source data are provided as a Source Data file.

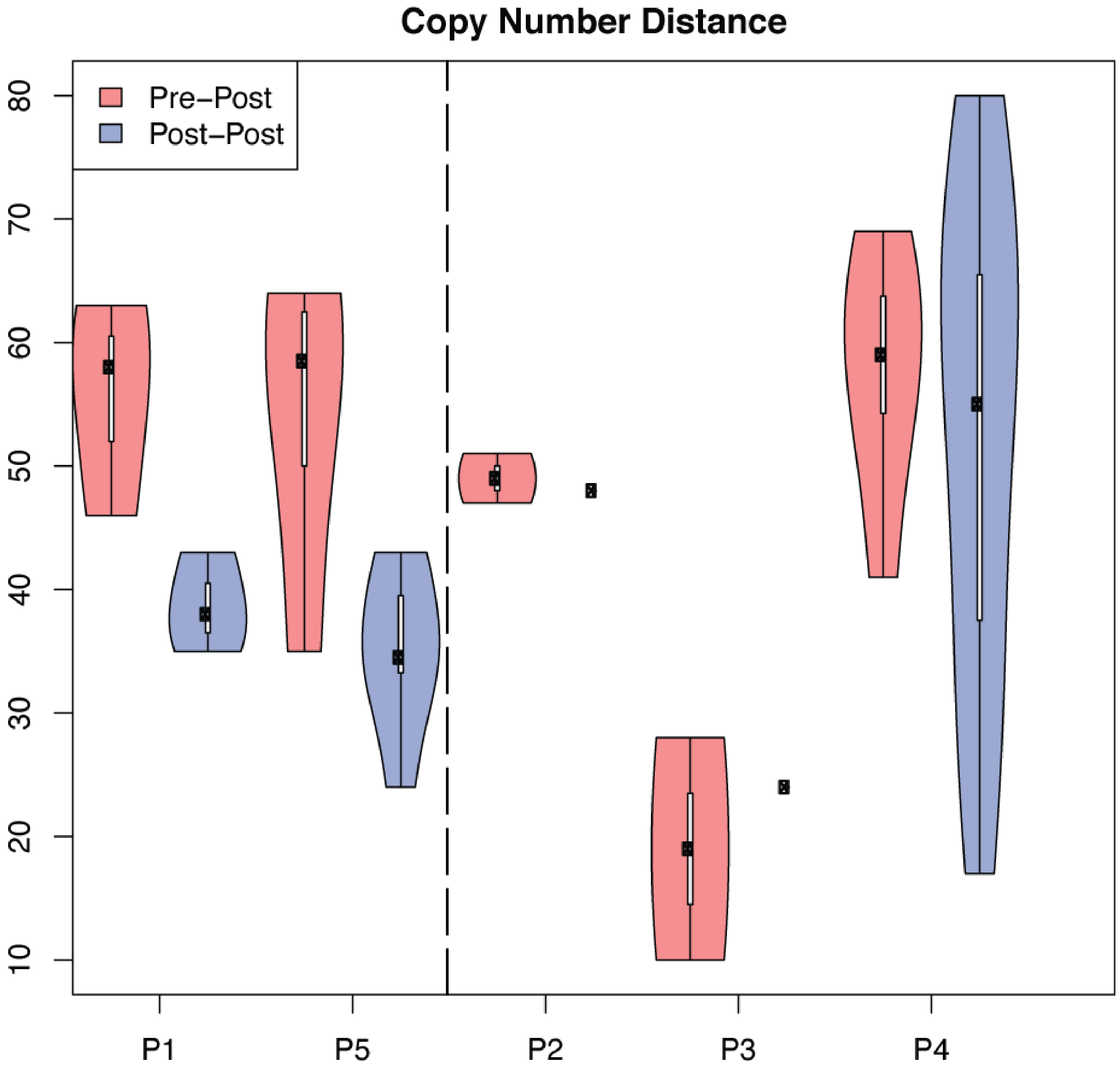


**Supplementary Figure 10. Increased post-treatment copy number similarity after clonal replacement** The pairwise copy number distances inferred with MEDICC were higher in pairs of pre-treatment – post-treatment samples than in pairs of post-treatment – post-treatment samples in P1 and P5 (which underwent clonal replacement), but were similar pre-post to post-post in P2, P3, and P4 (which did not). Source data are provided as a Source Data file.

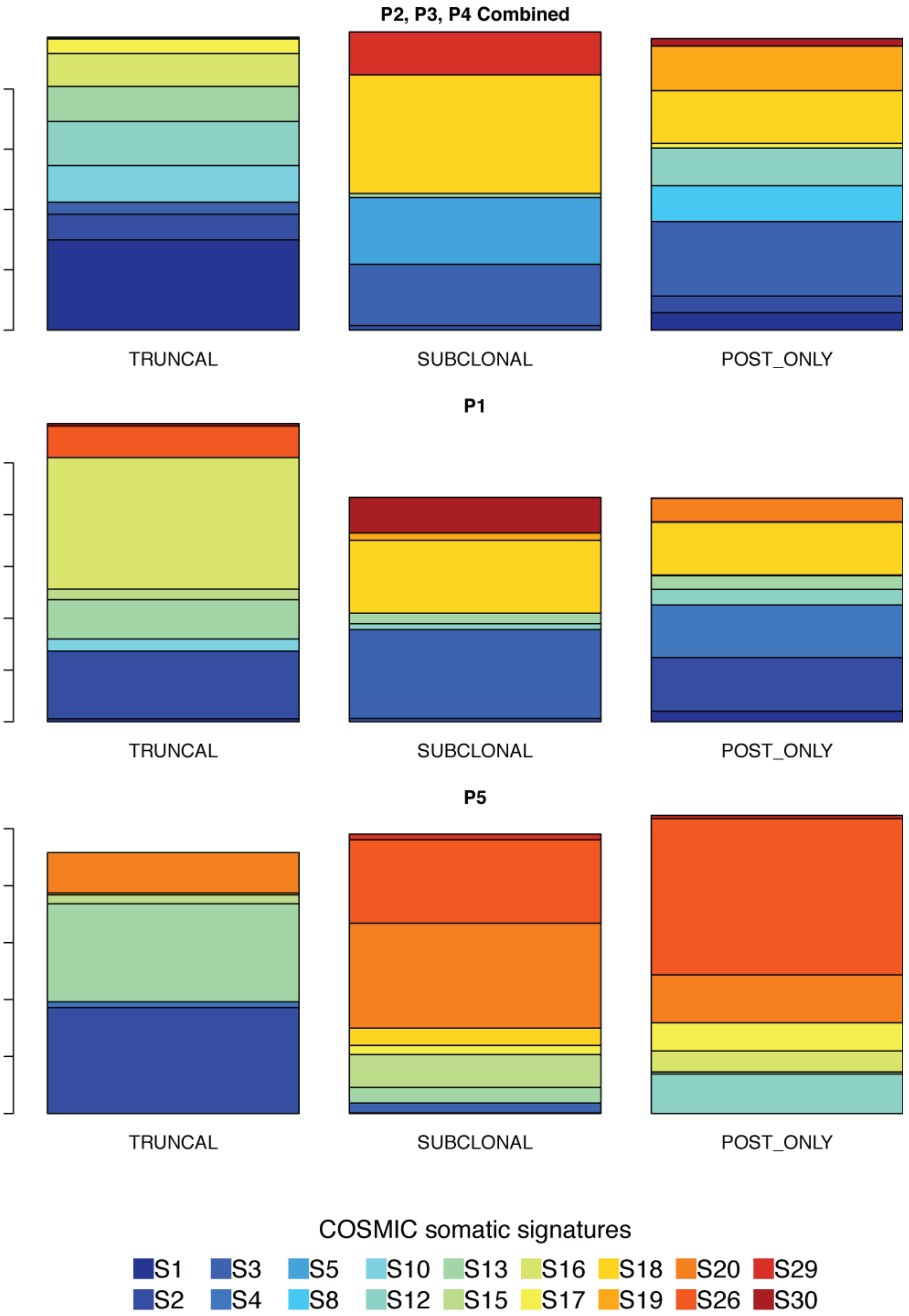


**Supplementary Figure 11. Somatic mutational signatures across mutation groupings** We defined non-synonymous mutations as truncal, subclonal, or post-only (see **Methods**) and compared the COSMIC mutational signatures present in each category. Truncal mutations appeared to result from a different set of mutational processes than subclonal and post-treatment mutations, while subclonal and post-treatment mutations shared a similar set of mutational signatures. Source data are provided as a Source Data file.

**Supplementary Table 1. Percentage of mutations identified as clonal in the first region that were later determined to be rare in additional tumor regions**

| | Addition of 2nd region | Addition of 3rd region | Addition of 4th region |
|---|---|---|---|
| **P6** | 24.10% | 12.80% | 9.24% |
| **HER2_2** | 13.26% | 4.44% | 4.09% |
| **ER_3** | 31.50% | 3.40% | |
| **TN_1** | 15.30% | 1.02% | |
| **TN_7** | 3.94% | 1.63% | 0.32% |

**Supplementary Table 2. Characteristics of analyzed and excluded treated HER2+ tumors**

| | Analyzed (N=5) | Excluded (N=15) |
|---|---|---|
| **ER-status** | | |
| Positive | 1 (20%) | 9 (60%) |
| Negative | 4 (80%) | 6 (40%) |
| **Grade** | | |
| 2 | 0 | 1 (8%) |
| 3 | 5 (100%) | 12 (92%) |
| Unknown | 0 | 2 |
| **Clinical stage** | | |
| IIB | 0 | 2 (14%) |
| IIIA | 2 (40%) | 3 (21%) |
| IIIB | 3 (60%) | 6 (43%) |
| IIIC | 0 | 3 (21%) |
| Unknown | 0 | 1 |
| **Treatment** | | |
| TCH | 4 (80%) | 3 (21%) |
| AC-TH | 0 | 10 (71%) |
| Other | 1 (20%) | 1 (7%) |
| Unknown | 0 | 1 |
| **Post-treatment tumor size (cm)*** | 4.3 (sd 1.7) | 1.7 (sd 1.7) |

* $P$ = 0.02 (two-sided $t$ test)
