# Supplementary material for: Clonal replacement and heterogeneity in breast tumors treated with neoadjuvant HER2-targeted therapy
Source: Nat Commun. 2019 Feb 8;10:657. doi: 10.1038/s41467-019-08593-4 (PMC6368565; doi:10.1038/s41467-019-08593-4)
Supplement: Supplementary file 3 — Description of Additional Supplementary Files [file 41467_2019_8593_MOESM3_ESM.pdf]

Description of Additional Supplementary Files

File Name: Supplementary Data 1

Description: Characteristics of tumors analyzed in this study

File Name: Supplementary Data 2

Description: Somatic single nucleotide variants and cancer cell fractions for untreated and treated tumors sequenced in this study

File Name: Supplementary Data 3

Description: Protein-altering mutations and indels in driver and target genes

File Name: Supplementary Data 4

Description: Protein-altering mutations in post-treatment clones

File Name: Supplementary Data 5

Description: Copy number changes in post-treatment clones
