# Supplementary material for: Clonal replacement and heterogeneity in breast tumors treated with neoadjuvant HER2-targeted therapy
Source: Nat Commun. 2019 Feb 8;10:657. doi: 10.1038/s41467-019-08593-4 (PMC6368565; doi:10.1038/s41467-019-08593-4)
Supplement: Supplementary file 10 — Reporting Summary [file 41467_2019_8593_MOESM10_ESM.pdf]

nature research

Corresponding author(s): Christina Curtis

Reporting Summary

Nature Research wishes to improve the reproducibility of the work that we publish. This form provides structure for consistency and transparency in reporting. For further information on Nature Research policies, see Authors & Referees and the Editorial Policy Checklist.

Statistical parameters

When statistical analyses are reported, confirm that the following items are present in the relevant location (e.g. figure legend, table legend, main text, or Methods section).

n/a	Confirmed	
☐	☒	The exact sample size (n) for each experimental group/condition, given as a discrete number and unit of measurement
☐	☒	An indication of whether measurements were taken from distinct samples or whether the same sample was measured repeatedly
☐	☒	The statistical test(s) used AND whether they are one- or two-sided<br>*Only common tests should be described solely by name; describe more complex techniques in the Methods section.*
☒	☐	A description of all covariates tested
☒	☐	A description of any assumptions or corrections, such as tests of normality and adjustment for multiple comparisons
☐	☒	A full description of the statistics including central tendency (e.g. means) or other basic estimates (e.g. regression coefficient) AND variation (e.g. standard deviation) or associated estimates of uncertainty (e.g. confidence intervals)
☒	☐	For null hypothesis testing, the test statistic (e.g. F, t, r) with confidence intervals, effect sizes, degrees of freedom and P value noted<br>*Give P values as exact values whenever suitable.*
☒	☐	For Bayesian analysis, information on the choice of priors and Markov chain Monte Carlo settings
☒	☐	For hierarchical and complex designs, identification of the appropriate level for tests and full reporting of outcomes
☒	☐	Estimates of effect sizes (e.g. Cohen's d, Pearson's r), indicating how they were calculated
☐	☒	Clearly defined error bars<br>*State explicitly what error bars represent (e.g. SD, SE, CI)*

Our web collection on statistics for biologists may be useful.

Software and code

Policy information about availability of computer code

Data collection	No software was used for data collection.

Data analysis

1. For mutation calling: Mutect version 1.1.7, url https://github.com/broadinstitute/mutect
2. For variant filtering and rescuing: Variant Assurance Pipeline (our laboratory), url https://github.com/cancersysbio/VAP
3. For insertion/deletion calling: Strelka version 1, url https://sites.google.com/site/strelkasomaticvariantcaller/home
4. For local copy number and purity: TitanCNA, url https://bioconductor.org/packages/release/bioc/html/TitanCNA.html
5. For copy number heterogeneity, MEDICC, url http://www.markowetzlab.org/software/MEDICC.php
6. For mutational clustering, PyClone version 0.13.0, url https://github.com/aroth85/pyclone
7. For gene functional annotation, Polyphen-2, url http://genetics.bwh.harvard.edu/pph2/ and Cancer Genome Interpreter, url https://www.cancergenomeinterpreter.org
8. For somatic signatures, Somatic Signatures package https://bioconductor.org/packages/release/bioc/html/SomaticSignatures.html
9. For spatial modeling, Virtual Tumor Evolution (our laboratory), url https://github.com/cancersysbio/VirtualTumorEvolution and our modifications url https://github.com/cancersysbio/BreastCancerITH
10. For Approximate Bayesian Computation, ABC package, url https://cran.r-project.org/web/packages/abc/index.html
11. The code we used to select high confidence mutations and calculate heterogeneity in our tumors is available on github, url https://github.com/cancersysbio/BreastCancerITH

For manuscripts utilizing custom algorithms or software that are central to the research but not yet described in published literature, software must be made available to editors/reviewers upon request. We strongly encourage code deposition in a community repository (e.g. GitHub). See the Nature Research guidelines for submitting code & software for further information.

Data

Policy information about availability of data

All manuscripts must include a data availability statement. This statement should provide the following information, where applicable:
- Accession codes, unique identifiers, or web links for publicly available datasets
- A list of figures that have associated raw data
- A description of any restrictions on data availability

Whole-exome sequencing data generated for this study are deposited at the European Genotype Phenotype Archive (EGA) at EGAD00001004306. Data from previously published studies are available at: EGAS00001002153, EGAD00001000965, EGAD00001000898, EGAD00001001394, EGAD00001000714, EGAD00001000900, EGAD00001000984, EGAD00001001113, EGAS00001002947, and EGAS00001002737. The source data underlying Figures 1, 3, 4b, and 5b-c, as well as Supplementary Figures 2-4, 6-7, and 9-11, are provided as a Source Data file.

Field-specific reporting

Please select the best fit for your research. If you are not sure, read the appropriate sections before making your selection.

☒ Life sciences ☐ Behavioural & social sciences ☐ Ecological, evolutionary & environmental sciences

For a reference copy of the document with all sections, see nature.com/authors/policies/ReportingSummary-flat.pdf

Life sciences study design

All studies must disclose on these points even when the disclosure is negative.

Sample size	The size of our cohort (n=15 primary tumors and n=5 treated tumors with multiple timepoints) was dictated by the availability of high-quality multi-region sampling from patient tumors. Our goal is to characterize tumor evolution and heterogeneity across this cohort of patients, and we are careful to draw only conclusions supported by our sample.
Data exclusions	Exclusions are described in detail in the manuscript: results (page 6), methods (page 11), and supplementary figure 5 and supplementary table 4. In brief, we excluded tumors with low coverage after read duplicate removal (suggesting low quality or degraded DNA samples), tumors with low post-treatment cellularity, and tumors where there was no multi-region sampling available. These tumors must be excluded because our methods of analysis would not be possible on them. We discuss the potential implications of these exclusions in the discussion section (page 10-11).
Replication	Our variant assurance pipeline has been developed to ensure accuracy of mutation calls from which we draw inferences. We are cautious to only use mutations of high quality and confidence. The accuracy of whole-exome sequencing to call mutations has been validated in previous studies (e.g. https://doi.org/10.1016/j.cell.2013.01.019). For this reason, we did not perform technical replicates of our mutation calls.
Randomization	Not applicable -- there were no prespecified groups or outcomes.
Blinding	Not applicable -- there were no prespecified groups or outcomes.

Reporting for specific materials, systems and methods

Materials & experimental systems

n/a	Involved in the study
☒	☐ Unique biological materials
☒	☐ Antibodies
☒	☐ Eukaryotic cell lines
☒	☐ Palaeontology
☒	☐ Animals and other organisms
☐	☒ Human research participants

Methods

n/a	Involved in the study
☒	☐ ChIP-seq
☒	☐ Flow cytometry
☒	☐ MRI-based neuroimaging

Human research participants

Policy information about studies involving human research participants

Population characteristics	Current diagnosis (tumor subtype, grade, stage, and treatment) information is available in Supplementary Table 4. For the treated tumors, the patients' ages at diagnosis and treatment ranged from 50-64. All patients (breast cancer) are female.
Recruitment	Archival human biospecimens (tumor tissue and matched normal tissue) were analyzed. Any patient whose core diagnostic biopsy and surgical tumor specimen were both stored at the University of Southern California was eligible for inclusion. We do not anticipate biases from this process of identification of biospecimens.
